# Evidence of Accelerated Evolution and Ectodermal-Specific Expression of Presumptive BDS Toxin cDNAs from *Anemonia viridis*

**DOI:** 10.3390/md11114213

**Published:** 2013-10-30

**Authors:** Aldo Nicosia, Teresa Maggio, Salvatore Mazzola, Angela Cuttitta

**Affiliations:** 1 Laboratory of Molecular Ecology and Biotechnology, National Research Council, Institute for Marine and Coastal Environment (IAMC-CNR), Detached Unit of Capo Granitola, Torretta Granitola 91021, Trapani, Italy; E-Mail: aldo.nicosia@iamc.cnr.it; 2 Institute for Environmental Protection and Research—ISPRA, Palermo 90143, Italy; E-Mail: teresa.maggio@isprambiente.it; 3 National Research Council, Institute for Marine and Coastal Environment (IAMC-CNR), Calata Porta di Massa, Napoli 80133, Italy; E-Mail: salvatore.mazzola@cnr.it

**Keywords:** *Anemonia viridis*, BDS peptides, accelerated evolution, molecular modelling, tissue-specific libraries, gene expression pattern

## Abstract

*Anemonia viridis* is a widespread and extensively studied Mediterranean species of sea anemone from which a large number of polypeptide toxins, such as blood depressing substances (BDS) peptides, have been isolated. The first members of this class, BDS-1 and BDS-2, are polypeptides belonging to the β-defensin fold family and were initially described for their antihypertensive and antiviral activities. BDS-1 and BDS-2 are 43 amino acid peptides characterised by three disulfide bonds that act as neurotoxins affecting Kv3.1, Kv3.2 and Kv3.4 channel gating kinetics. In addition, BDS-1 inactivates the Nav1.7 and Nav1.3 channels. The development of a large dataset of *A*. *viridis* expressed sequence tags (ESTs) and the identification of 13 putative BDS-like cDNA sequences has attracted interest, especially as scientific and diagnostic tools. A comparison of BDS cDNA sequences showed that the untranslated regions are more conserved than the protein-coding regions. Moreover, the *K*_A_/*K*_S_ ratios calculated for all pairwise comparisons showed values greater than 1, suggesting mechanisms of accelerated evolution. The structures of the BDS homologs were predicted by molecular modelling. All toxins possess similar 3D structures that consist of a triple-stranded antiparallel β-sheet and an additional small antiparallel β-sheet located downstream of the cleavage/maturation site; however, the orientation of the triple-stranded β-sheet appears to differ among the toxins. To characterise the spatial expression profile of the putative BDS cDNA sequences, tissue-specific cDNA libraries, enriched for BDS transcripts, were constructed. In addition, the proper amplification of ectodermal or endodermal markers ensured the tissue specificity of each library. Sequencing randomly selected clones from each library revealed ectodermal-specific expression of ten BDS transcripts, while transcripts of BDS-8, BDS-13, BDS-14 and BDS-15 failed to be retrieved, likely due to under-representation in our cDNA libraries. The calculation of the relative abundance of BDS transcripts in the cDNA libraries revealed that BDS-1, BDS-3, BDS-4, BDS-5 and BDS-6 are the most represented transcripts.

## 1. Introduction

Sea anemones are generally poisonous animals that spend most of their lives in a sessile form; hence, capturing activities and defence mechanisms are strongly associated with toxin production [1]. The sea anemone *Anemonia viridis* is a widespread and extensively studied Mediterranean species [2,3,4,5,6], from which a large number of polypeptide toxins, including sodium and potassium ion channel modulators or blockers, as well as Kunitz-type protease inhibitors, have been isolated [7].

The K^+^ channel-blocking toxins can be grouped into different classes (type 1–4) based on the number of amino acid residues, molecular structure and target [8,9]. BDS peptides belong to the type 3 class, which includes peptides of various origins, such as APETx1 and APETx2 from *Anthopleura elegantissima* [10] or Am-II from *Antheopsis maculata* [11]. The first members of this class, BDS-1 and -2, were isolated 15 years ago and were originally described as blood pressure-reducing substances with antiviral activities [12]. BDS-1 and BDS-2 are 43 amino acid, cysteine-rich polypeptides characterised by three disulfide bonds. The peptides act on K^+^ channels containing Kv3 subunits, such as Kv3.1, 3.2, and 3.4; however, these peptides do not block Kv1.2, 1.3, 1.4, 1.5, 2.2, 4.2 or 4.3 K^+^ channels [13,14]. Ion channel inhibition develops as a result of the modification of Kv3 gating kinetics and not by the direct blockage of the channel pore [15]. Although BDS toxins were previously considered ineffective on sodium channels, BDS-1 was recently shown to strongly inactivate Nav1.7 channels and weakly inhibit Nav1.3 channels [16]. In addition, BDS-1 was also found to prevent neuronal death mediated by β-amyloid peptide through inhibition of Kv3.4-generated current [17].

Recently, the development of high-throughput sequencing technologies associated with digital transcriptome platforms has allowed the discoveries of novel members of known classes of toxins as well as novel peptide structures previously unknown in cold water sea anemones, such as *Bolocera tuediae* and *Hormathia digitata* [18].

Because toxins from natural venoms are a source of considerable scientific and diagnostic tools, the development of a large dataset of *A*. *viridis* ESTs [19] has attracted much interest. A search for toxin-encoding transcripts using a single residue distribution analysis (SRDA) in the EST database retrieved 12 putative BDS-like cDNA sequences (BDS-3 to BDS-14) in addition to BDS-1 [20].

In the present work, an open reading frame (ORF) for an additional putative BDS homolog (BDS-15) was recovered from the *A*. *viridis* EST collection, and *in silico* studies were performed on the peptide homologs constituting the arsenal of BDS toxins.

A comparison of BDS cDNA sequences showed that the untranslated regions (UTR) are more conserved than the protein-coding regions, which suggests mechanisms for accelerated evolution. A phylogenetic analysis and protein homology modelling were used to investigate the evolutionary relationships among the toxins affecting K^+^ channels and to establish conserved structural motifs. Furthermore, the efficient separation of the ectoderm from the endodermal layers of sea anemones enabled us to construct BDS tissue-specific cDNA libraries. Tissue expression profiles of the BDS toxins were analysed, and an ectodermal-restricted expression pattern emerged.

## 2. Results and Discussion

### 2.1. *A*. *viridis* BDS cDNA Characterisation

**Figure 1:**
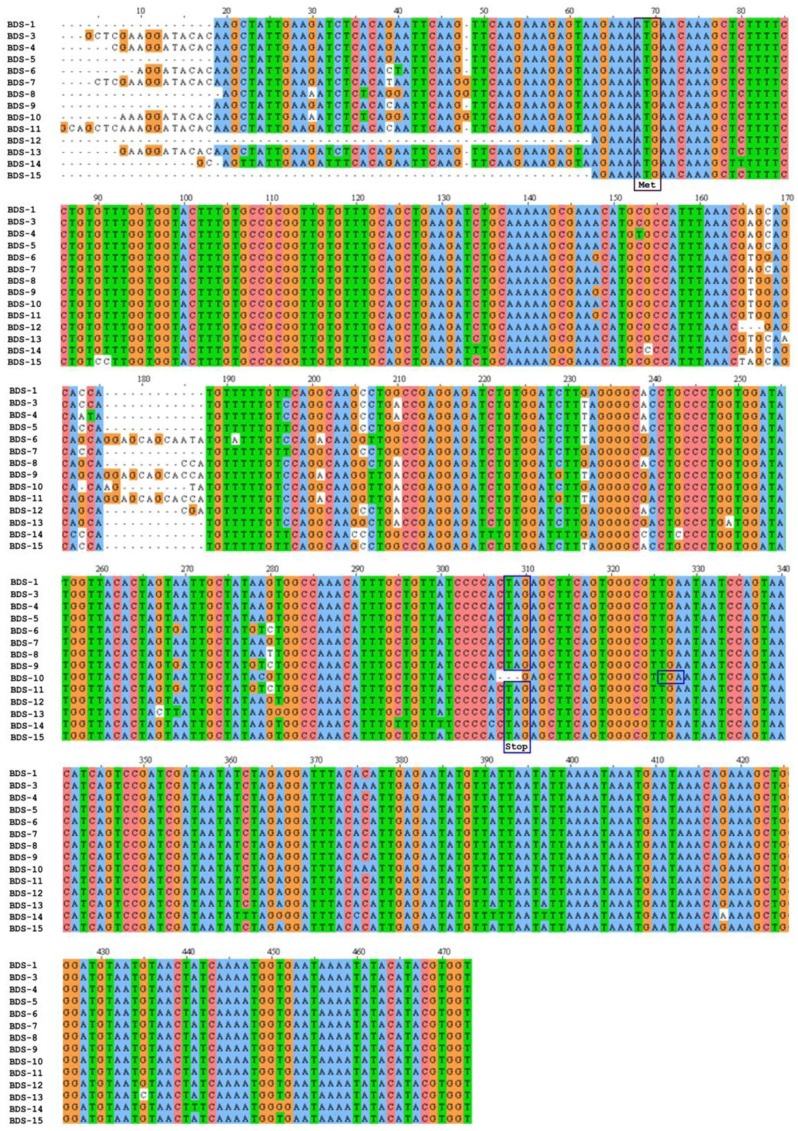
Multiple sequence alignment of BDS cDNAs from *A*. *viridis*. The cDNAs were aligned using ClustalW2. Dashes indicate gaps introduced to maximise the alignment. The ATG start codon is indicated by a purple box, while in-frame stop codons (TAG or TGA) are boxed in blue. The numbering of nucleotides refers to the alignment.

The cDNAs encoding the BDS toxins described previously [20] and BDS-15 were found in the EST database of the sea anemone *A*. *viridis*. A multiple sequence alignment was constructed to compare cDNAs encoding BDS peptides (Figure 1) and accession numbers for the ESTs are provided in Table 1. The open reading frames (ORFs) of the BDSs ranged from 231 to 249 bp, which encode proteins of 76 to 82 amino acids (Table 1). The stop codon was usually TAG, except for BDS-10, where it was replaced by TGA.

**Table 1:** BDS peptides in *A*. *viridis*.

Accession Number	Toxin	ORF length ^a^	Protein length ^b^	Molecular weight ^c^	pI ^d^
FK728690	BDS-1	231	76	8342.8	9.02
FK744472	BDS-3	231	76	8444.9	8.86
FK722457	BDS-4	231	76	8489.0	8.86
FK720902	BDS-5	231	76	8376.9	9.02
FK754940	BDS-6	243	80	8671.2	8.14
FK736435	BDS-7	231	76	8356.8	8.86
FK723172	BDS-8	234	77	8427.9	8.68
FK725608	BDS-10	249	82	9041.5	8.68
FK740326	BDS-11	243	80	8765.2	7.52
FK736010	BDS-12	231	76	8470.0	9.02
FK752236	BDS-13	231	76	8394.8	8.44
FK745823	BDS-14	231	76	8272.7	8.96
FK725211	BDS-15	231	76	8317.8	8.93

^a^ Length of cDNA, stop codon is included; ^b^ Length (No. of amino acids) of the deduced polypeptide; ^c^ Molecular weight of the deduced polypeptide in Dalton; ^d^ Isoelectric point of the deduced polypeptide.

BDS cDNA sequences exhibited high sequence identity in the ORFs, as well as in the 3′ and 5′ untranslated regions (UTRs) (Figure 1). In particular, BDS-1 displayed the highest sequence similarity with BDS-3, BDS-5, BDS-7, BDS-12 and BDS-15 (99% identity). Additionally, BDS-1 showed identity ranging from 98% to 96% with BDS-4, BDS-6, BDS-8, BDS-9, BDS-10, BDS-11 and BDS-13, while the lowest similarity was achieved comparing BDS-1 with BDS-14 (94% identity). A comparison of cDNAs encoding BDS-9 and BDS-11 showed that the two putative toxins are identical, except for a shorter 5′ UTR in the BDS-9 EST sequence; consequently, BDS-9 and BDS-11 were considered one (and indicated as BDS-11) for all subsequent computational and experimental analyses.

BDS-7, BDS-8 and BDS-10 EST sequences each contain a single nucleotide insertion upstream of the start codon (ATG); moreover, a few nucleotide substitutions were found in the same regions.

Compared with the other cDNAs, BDS-6, BDS-8, BDS-10, and BDS-11 contained insertion elements (12-bp insertion in BDS-6 and BDS-11, 4-bp insertion in BDS-10 and 3-bp insertion in BDS-8) mapping downstream of the start codon and located at a position between 176 and 187 in the alignment. A deletion of the BDS-10 ORF abrogates the conserved stop codon (TAG), causing the continuation of protein synthesis until the downstream stop codon (TGA) is encountered. Consequently, BDS-10 has the longest ORF of all the ESTs.

**Figure 2:**
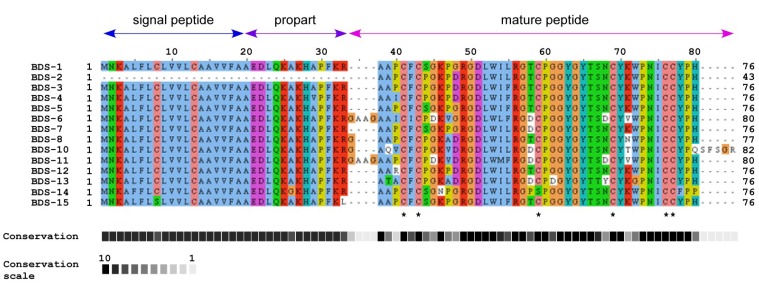
Protein sequence alignment of BDS peptides from *A*. *viridis* performed with ClustalW2. Below the sequences, asterisks mark cysteine residues located at the same positions.

A total of 473 sequenced base pairs were aligned, including the coding and non-coding regions, and after trimming, nucleotide sequence analyses were conducted on the region between positions 68 and 473 in the alignment. The sequence analysis revealed that 18 sites showed alignment gaps; 53 sites were variable, and 19 of these sites were parsimony-informative. Considering the regions between positions 68 and 307 of the alignment 42 sites were variable, and 18 were parsimony-informative; while, between positions 68 and 325 of the alignment, 43 sites were variable and 18 were parsimony informative. The protein sequence alignment contains 85 positions (Figure 2). Considering all the alignments, 24 amino acid sites were variable, and 9 of these sites are parsimony-informative.

To obtain further information about the evolution of BDS sequences, the number of nucleotide substitutions per site (*K*_N_) in the UTR regions and the number of nucleotide substitutions per synonymous site (*K*_S_) and non-synonymous site (*K*_A_) in the protein-coding region were calculated for the BDS cDNA sequences. Based on the neutral theory of molecular evolution [21], the *K*_A_ value never exceeds the *K*_S_ value. If *K*_A_ were to exceed *K*_S_, *K*_A_/*K*_S_ > 1 would result and serve as evidence that positive selection had acted to change the protein-coding region. The *K*_N_/*K*_S_ ratios were less than 1, while the *K*_A_/*K*_S_ ratios calculated for all pairwise comparisons ranged from 0.98 to 4.4, with an average value of 1.81. This pattern is indicative of strong positive selection for amino acid replacements because it implies that the rate of non-synonymous changes exceeds that which can be explained solely by a neutral substitution rate. These emerging features reflect the evolutionary mechanisms described previously for some toxins affecting voltage-gated sodium channels from sea anemones [2,3], isozymes of snake venom [22,23], and toxins from scorpions [24], which have evolved in an adaptive manner under positive Darwinian selection (“accelerated evolution”). Hence, selection promotes the fixation of non-synonymous substitutions and accelerates the diversification of related sequences.

### 2.2. Comparison of BDS Amino Acid Sequences

The characteristics of the analysed BDS protein sequences are summarised in Table 1. The predicted proteins have an estimated molecular mass ranging from 8317.8 (BDS-15) to 9041.5 Da (BDS-10) and a theoretical pI between 8.14 and 9.02.

The deduced BDS neurotoxins are synthesised as inactive precursors with a putative N-terminal leader peptide (residues 1–19), a propart of 14 residues, a cleavage site for proteolytic activation, and the mature toxins (Figure 2). The propart is composed primarily of polar and negatively charged amino acids and is cleaved upon toxin maturation. In addition, the propart has been proposed to have a role in intracellular sorting and delivery to target the toxin to the nematocyst [25,26]. However, the presence of a propart is not limited to neurotoxin peptides because it has also been identified in cytolysins from *Actineria villosa* [27]. Although the BDS peptide sequences are highly similar, BDS-6, BDS-8, BDS-10 and BDS-11 contain one or four additional residues at the *N*-termini of their mature peptides. BDS-10 also contains an extra five residues at the *C*-terminus due to the absence of the otherwise highly conserved stop codon (TAG).

BDS-15 differs from all other BDS peptides because it lacks the highly conserved dyad K^32^R^33^, and Cys is substituted with Ser at position 8 in the leader peptide. This result is anomalous if compared with previous reports on sea anemone toxins containing a tandem Lys-Arg at the C-terminal end of the propart [18,25,26,28]. In this context, BDS-15 appears to resemble other proteins, including SNTX [29], Galectin-3 [30], Hk2a [31] and AvTX-20 [27] toxins. Consequently, a different secretory mechanism, without the involvement of the endoplasmic reticulum or Golgi apparatus, which was hypothesised for such proteins [32], might also be extended to BDS-15. The processing required for toxin activation may not be essential for cellular activity, as demonstrated previously for the pertussis toxin [33]. Nevertheless, the effective functionality of the putative BDS-15 toxin remains unaddressed.

### 2.3. Sequence Similarity with Other Peptide Toxins

**Figure 3:**
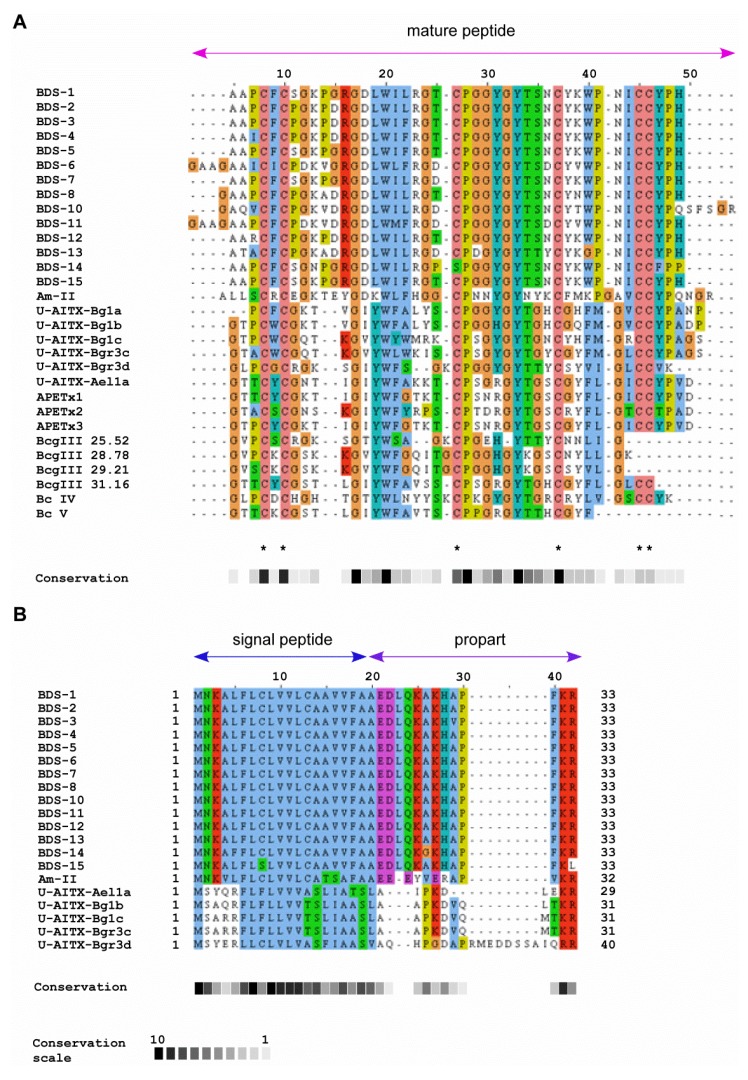
Multiple sequence alignment of *A*. *viridis* BDSs with other sea anemone K^+^ channel toxins. (**A**) Mature peptide alignment. Cys residues are marked with an asterisk; (**B**) Alignment of signal peptides and proparts. Abbreviations, species, and accession numbers are listed in Table 2 (see Experimental Section).

A basic local alignment search tool (BLASTp) analysis indicated that all BDS toxins share moderate to high similarity with other sea anemone toxins active on voltage-dependent K^+^ channels. Mature BDS peptides displayed identity ranging from 40% to 46% with the Am II toxin of *Antheopsis maculata*, U-AITX-Bg1, and Bgr3 peptides from *Bunodosoma granuliferum*. Significant similarity (40% to 47% identity) with the APETx-like peptides from *Anthopleura elegantissima* and Bcg and Bc toxins (identity ranging from 39% to 42% and from 40% to 46%, respectively) from *Bunodosoma cangicum* was also shared with the mature BDS toxins (Figure 3A).

A multiple sequence alignment was also constructed for the signal peptides and proparts of BDS toxins and other sea anemone toxins for which full-length sequences have been reported (Figure 3B). The BDS signal peptides and proparts displayed highest sequence similarity (64% identity) with the 32 amino acid Am-II precursor from *A*. *maculata*. With the exception of BDS-15, the subtilisin-like protease cleavage site (Lys-Arg or Arg-Arg) is conserved among all the sequences; additionally, the signal peptides and proparts are related.

**Table 2:** Kv channel toxins from sea anemones, accession numbers and related references.

Species	Toxin	GenBank accession number	References
*Bunodosoma cangicum*	Bcg III 29.21	P86464	Zaharenko *et al*. 2008 [34]
	Bcg III 25.52	P86463	
	Bcg III 28.78	P86462	
	Bcg III 31.16	P86461	
*Bunodosoma granuliferum*	U-AITX-Bg1a	CCC86602	Rodriguez *et al*. 2012 [35]
	U-AITX-Bg1b	CCC86603	
	U-AITX-Bg1c	CCC86604	
	U-AITX-Bgr3c	CCC86605	
	U-AITX-Bgr3d	CCC86606	
*Bunodosoma caissarum*	BcIV	P86470	Oliveira et al. 2006 [36]
	BcV	P84919	Zaharenko, A.Z. [37]
*Antheopsis maculata*	Am II	P69930	Honma *et al*. 2005 [11]
*Anthopleura elegantissima*	APETx1	P61541	Diochot *et al*. 2003 [10]
	APETx2	P61542	
	APETx3	B3EWF9	Peigneur, S. [38]
	U-AITX-Ael1a	FG392547 *	Richier *et al*. 2008 [39]

* FG392547 refers to an EST derived by Richier *et al*. (2008) [39]; The deduced amino acid was reported by Rodriguez *et al*. 2012 [35] as U-AITX-Ael1; however, no accession number has been associated.

### 2.4. Phylogenetic Analysis

**Figure 4:**
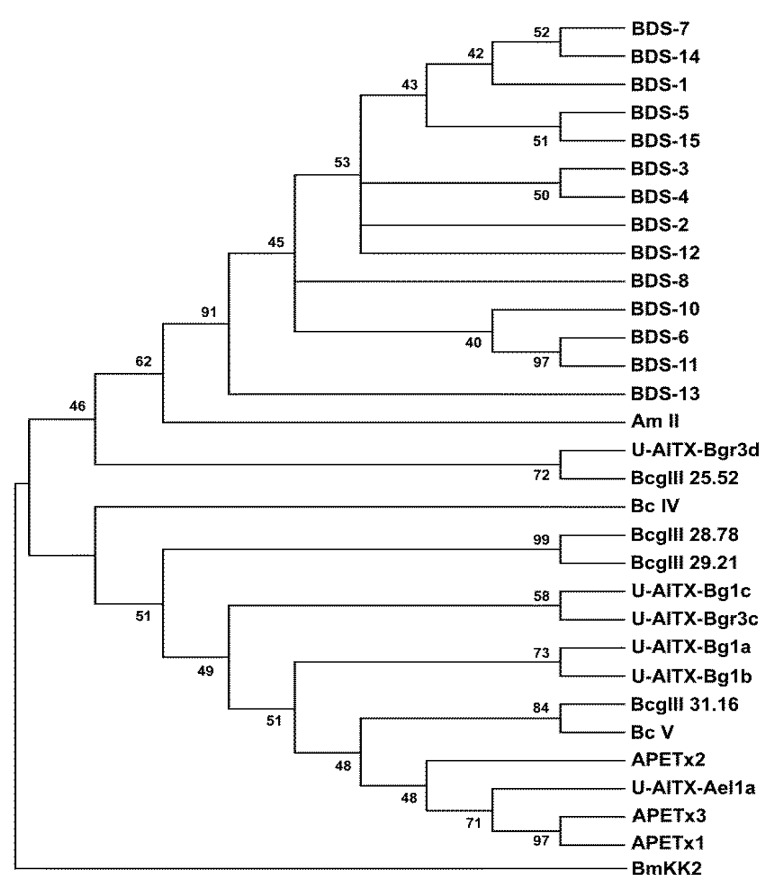
Neighbour-Joining (NJ) phylogenetic tree based on 14 BDS amino acid sequences and other cnidarian Kv channel toxins from GenBank using the *p-*distance model. Toxin BmKK2 (Q95NK7) from *Mesobuthus martensii* was used as outgroup. Internal branches were assessed using 1000 bootstrap replications. Branches corresponding to positions reproduced in less than 40% bootstrap replicates are collapsed.

To investigate the evolutionary relationships among the toxins affecting K^+^ channels, a phylogenetic tree was constructed using the 14 mature BDS toxins and the mature protein sequences of 16 sea anemone neurotoxins (Figure 4).

Phylogenetic analysis displayed the presence of two well-defined clades; the first clade represents the 14 BDS toxins from *A*. *viridis*, the Am-II peptide from *A*. *maculata*, the U-AITX-Bgr3d from *Bunodosoma granulifera* and Bcg III 25.52 from *B*. *cangicum*. The second clade groups the BDS-like peptides, as inferred from Pfam and InterPro annotations, from *A*. *elegantissima* (U-AITX-Ael1a, APETx1, APETx2 and APETx3), *B*. *cangicum* (Bcg III 28.78, Bcg III 29.21, and Bcg III 31.16), *B*. *caissarum* (BcIV and BcV), and *B*. *granulifera* (U-AITX-Bg1a, U-AITX-Bg1b, U-AITX-Bg1c and U-AITX-Bgr3c).

### 2.5. Homology Modelling

**Figure 5:**
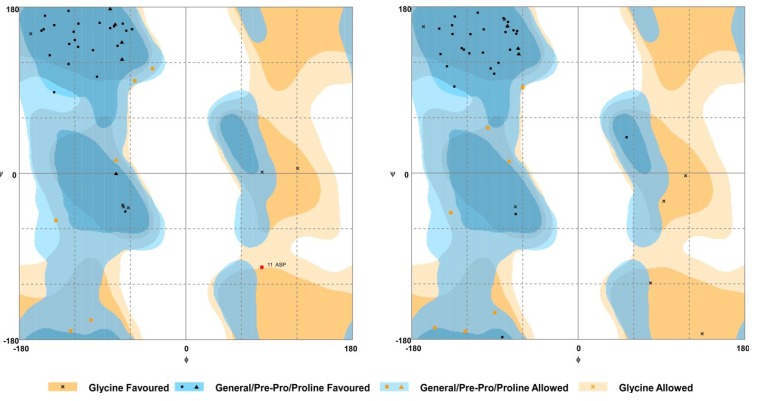
Representative Ramachandran maps of the predicted 3D model of two BDS peptides (BDS-3 on the left; BDS-6 on the right). Each amino acid residue is shown as a symbol in a graph of φ *vs*. ψ. For further details, refer to the keys beneath the maps.

To establish conserved structural motifs, the 3D structures of the BDS toxins were predicted by homology modelling using BDS-I (PDB ID 1bds) as the template, and the generated models were validated by assessing a Ramachandran plot (Figure 5). The results of these analyses showed that the percentage of residues in the favoured/allowed region ranged from 90% to 95% among all the predicted models.

**Figure 6:**
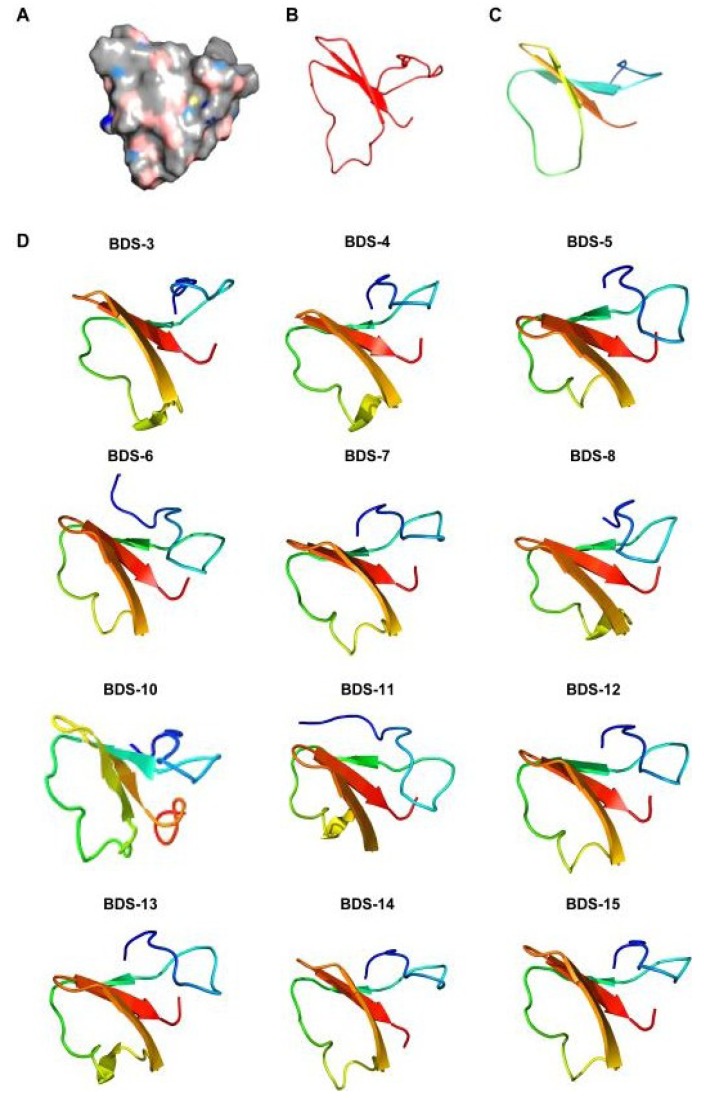
Homology modelling of BDS toxins. The models were built based on the NMR structure of the mature form of BDS-1. (**A**) BDS-1 surface in an electrostatic potential model; (**B**,**C**) Structural elements of BDS-1 and BDS-2; (**D**) Structure predictions of the BDS toxins. The colour transition from blue to red represents the transition from the *N*- to *C*-terminus.

Modelling analysis predicted β-strand configurations for the mature BDS toxins (Figure 6), as previously described for the mature BDS-1 and BDS-2 toxins [40,41], and α-helical structures in the leader peptides and proparts (data not shown).

The mature toxins are usually organised in a triple-stranded antiparallel β-sheet. Loops connecting the strands of the antiparallel β-sheet and an additional antiparallel β-strand located downstream of the cleavage/maturation site are also present. Despite the observed residue substitutions and the insertion of the tripeptide A^36^GA^38^ into BDS-6 and BDS-11 as well as the pentapeptide S^78^FSGR^82^ into BDS-10, a comparison of BDS structures reveals that the toxins possess grossly similar 3D structures. However, the orientation of the triple-stranded β-sheet seems to be different and change among the toxins. Thus, such changes might influence the activity and specificity of the neurotoxins constituting the BDS arsenal of *A*. *viridis*.

### 2.6. Tissue-Specific Gene Expression Pattern

Real-time polymerase chain reaction (RT-PCR) represents a robust technique with which to study the expression profile of a specific gene. However, due to the low variability among BDS cDNA sequences, it was not possible to design a suitable primer set to selectively amplify the sequence variants. Instead, sequencing randomly selected clones from cDNA libraries is a useful alternative to investigate genes and gene expression patterns in a tissue of interest [42].

To analyse the tissue distribution and expression profiles of BDS toxins at the transcriptional level, total RNA was isolated from ectodermal- and endodermal-derived tissues. To monitor contamination of ectodermal or endodermal extracts, the transcripts for an isoform of carbonic anhydrase 2 (CA2-M) and hydroxymethylglutaryl-CoA reductase (HMG-CoA reductase) [43] were reverse transcribed and amplified in all samples as endoderm and ectoderm markers, respectively. CA2-M and HMG-R amplicons were usually detected after 20 cycles in endodermal and ectodermal tissues, respectively, and limited cross-contamination was evident after 30 cycles of amplification. Additionally, CA2-M and HMG-R transcripts were co-expressed in the pharynx and muscles (data not shown).

To avoid biases that could result in the loss of certain species of BDS transcripts, tissue-specific cDNA libraries enriched for BDS transcripts were generated. A limited number of amplification cycles were performed to minimise the amount of contaminating cDNA sequences that could impair the results and to ensure that high target concentrations did not reach saturation.

**Figure 7:**
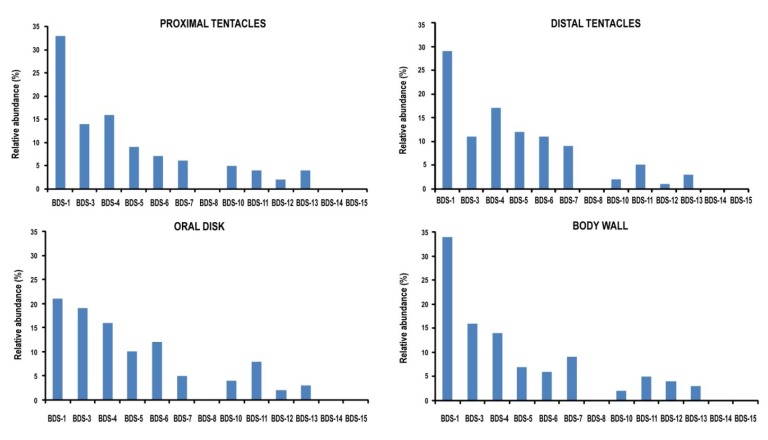
Relative frequencies of BDS clones in ectodermal tissues of *A*. *viridis*. Variations in the number of clones in the library were considered a measure of gene expression.

Tissue-specific cDNA clones were screened by colony PCR, and 200 independent clones per tissue were sequenced. BDS transcripts were detected in ectodermal tissue from the distal and proximal tentacles (the tentacle’s base), body wall, and oral disk, with different relative abundances (Figure 7). In total, transcripts of ten BDS representatives were identified, while BDS-8, BDS-14 and BDS-15 were not retrieved. In contrast, no BDS transcripts were detected in the pharynx, gonads, and muscle tissues, although these tissues contain endodermal and ectodermal tissues. This result indicates that these transcripts are extremely underrepresented in our cDNA libraries, either due to low expression levels or a very restricted expression pattern. The ectodermal cells constituting the external epithelial layer of tentacles, body wall and oral disk exerts several functions including defence against predators and capturing and killing prey. The ectodermal cells of the pharynx, gonads, and muscles participate in different physiological functions, which include digestion, gamete development and movement processes. Thus, the presence of cells responsible for the active transcription and production of BDS neurotoxins is not required in these tissue areas. However, the limited number of cycles performed for the preparation of the cDNA libraries may have contributed to a slight underrepresentation.

The relative abundance of a clone was considered to be a proxy for transcriptional activation, and as shown in Figure 7, the levels of BDS-1 transcript in our ectodermal cDNA libraries were the highest in all the analysed tissues with a relative abundance ranging from 21% to 34%. According to the literature [20], BDS-1 is the most represented component of the family in *A*. *viridis*. Levels of expression from high to moderate were also shown for BDS-3, BDS-4, BDS-5 and BDS-6; conversely, the number of clones for BDS-7, BDS-10, BDS-11, BDS-12 and BDS-13 cDNAs was less abundant.

To validate the results, the relative abundances of BDS transcripts were compared to the number of ESTs encoding the BDS toxins in the *A*. *viridis* database, and a total of 49 EST sequences were collected. The cDNAs encoding BDS-3, BDS-4, BDS-5 and BDS-6 were well represented due to the presence of five or six EST sequences each; meanwhile, ESTs encoding BDS-7, BDS-10 and BDS-11 showed lower representation. The cDNAs encoding BDS-8, BDS-14 and BDS-15 were the least abundant (one EST encoding each toxin). This result may explain why no clones encoding these putative toxins were retrieved after sequencing our cDNA libraries.

## 3. Experimental Section

### 3.1. GenBank Accession Numbers

The BDS sequences were obtained from the *A*. *viridis* EST database [19] at the National Centre for Biotechnology Information (NCBI) under the following accession numbers: FK728690 (BDS-1), FK744472 (BDS-3), FK722457 (BDS-4), FK720902 (BDS-5), FK754940 (BDS-6), FK736435 (BDS-7), FK723172 (BDS-8), FK721972 (BDS-9), FK725608 (BDS-10), FK740326 (BDS-11), FK736010 (BDS-12), FK752236 (BDS-13) and FK745823 (BDS-14) [20]. The BDS-15 sequence, accession number FK725211, was extracted from the *A*. *viridis* EST database using the TBLASTN search tool.

### 3.2. Sequence and Phylogenetic Analyses

The nucleotide and deduced amino acid sequences of BDS cDNAs were compared with other known sequences, including the BDS-II toxin (P59084), using the BLAST algorithm and the ExPASy Bioinformatics Resource Portal. Nucleotide polymorphisms and substitution rates among the BDS ESTs were determined using DNAsp software [44] and Molecular Evolution Genetics Analyses (MEGA) software version 5.0 [45].

We computed the number of conserved, variable, and parsimony-informative sites. The number of nucleotide substitutions per site (*K*_N_) in the untranslated regions (UTRs) and the number of nucleotide substitutions per synonymous site (*K*_S_) and non-synonymous site (*K*_A_) in the protein-coding region were computed for all the pairwise comparisons of BDS sequences according to the method of Nei and Gojobori [46]. *K*_A_/*K*_S_ ratios were used to evaluate the selective forces acting on these proteins.

Signal peptides, functional sites and domains in the predicted amino acid sequences were predicted by the Simple Modular Architecture Research Tool (SMART) program, the InterPro database, the Pfam database, the PROSITE program, and the Eukaryotic Linear Motif resource (ELM) for Functional Sites in Proteins. Multiple sequence alignments were performed using the ClustalW 2.0 program at the European Bioinformatics Institute. Phylogenetic and molecular evolutionary analyses were conducted on the amino acid sequences of the mature BDS toxins and 16 cnidarian Kv channel toxins (Table 2) using the Neighbour-Joining (NJ) method in MEGA 5.0. Evolutionary distances were estimated using the *p-*distance model, and alignment gaps were deleted prior to calculation. Internal branch support was assessed using 1000 bootstrap replications. A phylogenetic tree was determined using the BDS toxins and other cnidarian neurotoxin sequences recovered in GenBank and reported in Table 2. The K^+^ channel blocker BmKK2 toxin (GenBank Accession Number: Q95NK7) from the venom of chinese scorpion *Mesobuthus martensii* was used as outgroup.

### 3.3. Homology Modelling

BDS 3D structures were reconstructed by homology modelling via the Protein Homology/analogY Recognition Engine 2.0 (Phyre 2) software [47], using the intensive modelling mode. Candidate structures for homology modelling were selected according to pairwise alignment and cysteine distribution. The NMR 3D solution structure of BDS-1 (PDB code: 1bds) was used as a template, and homology models were built for all of the sets of proteins. Validation of the structural protein models was performed by assessing the Ramachandran plots. Cycles of clash minimisation were also performed for the refinement of structures.

### 3.4. RNA Extraction and First-Strand cDNA Synthesis

Specimens of *A*. *viridis* were collected from the Capo Granitola coast (Torretta Granitola, Trapani, Italy) in the south of Sicily and maintained in a 200 L aerated seawater tank. Tentacles, oral disks, and body walls were cut lengthwise and scraped to separate the endodermal cell layer and the film of mesogloea from the ectodermal cells. The small size of the pharynx, muscles and gonads prevented the separation of ectodermal from endodermal tissue; consequently, RNA was isolated from both ectodermal and endodermal layers. Tissues were frozen in liquid nitrogen and ground into a fine powder using a tissue disruptor. The powder was dissolved in Trizol reagent (Invitrogen Corporation, Carlsbad, CA, USA), and further RNA purification steps were performed according to the manufacturer’s instructions. RNA concentrations and quality were verified by spectrophotometry (optical density (OD) at 260 nm), while RNA integrity was checked using a 1.5% agarose gel. The RNA was stored at −80 °C for future use. The extracted RNA (2 μg) was treated with RNA qualified 1 (RQ1) RNase-Free DNase (Promega, Madison, WI, USA) to remove any residual genomic DNA contamination, and DNase I was inactivated by adding 25 mM EDTA. First-strand cDNA was synthesised from 2 μg DNase I-treated total RNA samples using oligo(dT)_18_ and Superscript III (Invitrogen Corporation), following the manufacturer’s instructions. The cDNA mixture was stored at −20 °C until needed.

### 3.5. Tissue-Specific BDS cDNA Library Construction

RNA quality and cross-contamination were assessed by performing RT-PCR reactions using CA2-M and HMG-R specific primers (Table 3), and the expected bands were obtained.

**Table 3:** Oligonucleotide primers used in this study.

Primers	Sequences (5′–3′)	Amplicon size (bp)
BDS-F	GAAAATGAACAAAGCTCTTTCC	278–290
BDS-R	GATCGGACTGATGTTACTGG	
CA2-m-F	CTTTGGCGGCATTTCACTTG	129
CA2-m-R	GTGATTGGTTGGAGCCATCG	
HMG-F	AGTATGTGAAGCCATAGTGC	311
HMG-R	TAGTACCACCACCAACAGTC	

The cDNAs derived from the ectodermal and endodermal tissues were separately subjected to PCR reactions using a pair of primers (BDS-F and BDS-R) specifically chosen to ensure the amplification of all the BDS sequence variants in the EST database.

PCR reactions were performed using AmpliTaq Gold DNA polymerase (Applied Biosystems, Forster City, CA, USA) with the following conditions: Pre-denaturation at 94 °C for 4 min, 25 cycles of 94 °C for 30 s, 52 °C for 30 s, and 72 °C for 30 s, followed by elongation at 72 °C for 8 min.

Amplified products that were representative of BDS sequence variants in each studied tissue were analysed using a 1.4% agarose gel and subcloned into the pGEM*-*T Easy vector (Promega).

The resulting tissue-specific libraries were transformed into the *Escherichia coli* strain DH10B (Promega) and seeded onto 150 mm Luria-Bertani-Agar plates containing 100 μg/mL of ampicillin. Library screening was performed by colony PCR using the BDS-F and BDS-R primers.

From each library, 200 recombinant clones were picked and replicated, and the plasmid DNA was purified using a QIAprep Spin Miniprep Kit (QIAGEN, Tokio, Japan) and sequenced using T7 and SP6 primers on an ABI-3730 Genetic Analyser (Applied Biosystems). The relative abundance of a clone was computed by dividing the number of a specific BDS clone with the total number of sequenced clones per library.

## 4. Conclusions

Sea anemones are a well-known source of neurotoxins acting upon voltage-gated sodium and potassium ion channels [48]. *A*. *viridis*, the Mediterranean species of sea anemone, is unable to retract and is exposed to attack from predators, such as Nudibranchia. Moreover, *A*. *viridis* captures food actively and feeds on a wide spectrum of prey, including crustaceans and molluscs [49]. Thus, the chemical arsenal of *A*. *viridis* represents the preferred strategy to survive in such a habitat and the components of the produced neurotoxin cocktail are very copious [2,50].

In the present study, comparative analysis of BDS cDNA sequences allowed us to hypothesise that mechanisms of accelerated evolution occurred in the protein-coding region of BDS peptides. Additionally, although the 3D structure of BDS toxins remains grossly unchanged, the orientation of the triple stranded β-sheet appears to be modified among the models. Such diversity and variations may be responsible for the activity on K^+^ channels containing Kv3 subunits and also for the inactivation of Nav1.7 and Nav1.3 channels exerted by preparations of extracted BDS toxins [16].

Because the capture and killing of prey as well as mechanisms of defence and protection in sea anemones are closely related to toxin production, the presence of multiple variants generated by accelerated evolution could provide some benefit. The variety of putative BDS toxins produced by *A*. *viridis* could reflect the high environmental adaptability of the species. Previous studies showed that the evolution of toxin diversity is linked to the diet and ecology of a species [51,52]. Thus, similar mechanisms appear to have affected the diversity of BDS toxins, equipping the sea anemone with important systems for survival.

Herein, the spatial expression profile of the putative BDS cDNA sequences was also examined. A recent report has demonstrated that Type I neurotoxins from *Nematostella vectensis*, *A*. *elegantissima* and *A*. *viridis* are confined to nematocytes and ectodermal gland cells [53]. Despite phylogeny and different channel specificity, gene expression studies revealed that BDS transcripts were co-expressed in ectodermal tissue. Further studies focused on the identification of BDS toxin-synthesising cells will be helpful to gain further insight on mechanisms of toxin production and delivery.

In addition to their natural role in sea anemones, BDS toxins were considered of scientific interest in Central Nervous System (CNS) studies since their first characterisation. Several studies reported the use of such antagonists as a pharmacological tool to assign functional roles to channels in CNS neurons [54,55,56]. Because the Kv3 potassium channels are essential in CNS physiological activity and Kv3.4 subunits were shown to be involved in responses to chronic hypoxia [57] in Parkinson’s [55] and Alzheimer’s [58] diseases, selective targeting of the aforementioned subunits will have therapeutic utility in CNS disorders.

Recombinant DNA technology associated with functional bioassays could allow a specific BDS toxin to be produced in heterologous expression systems. This technology could lead to the development of new peptides with restricted activity as biopharmaceuticals for the treatment of neuronal diseases.
